# Investigating the role of uric acid and uric acid‐to‐creatinine ratio as a predictive factor of chronic obstructive pulmonary disease exacerbation in 2019

**DOI:** 10.1111/crj.13689

**Published:** 2023-08-29

**Authors:** Saman Barmehziar, Abbas Fadaii, Fariba Samadian, Ali Shakiba, Sogol Koolaji

**Affiliations:** ^1^ Department of Internal Medicine, Shahid Labbafinejad Hospital Shahid Beheshti University of Medical Sciences Tehran Iran; ^2^ Department of Pulmonology and Intensive Care Medicine, Shahid Labbafinejad Hospital Shahid Beheshti University of Medical Sciences Tehran Iran; ^3^ Department of Nephrology, Shahid Labbafinejad Hospital Shahid Beheshti University of Medical Sciences Tehran Iran; ^4^ Non‐Communicable Diseases Research Center, Endocrinology and Metabolism Population Sciences Institute Tehran University of Medical Sciences Tehran Iran

**Keywords:** chronic obstructive lung disease, chronic obstructive pulmonary disease (COPD), disease exacerbation, severity of disease, uric acid

## Abstract

**Introduction:**

Serum uric acid has been suggested as an independent marker of oxidative metabolism in chronic obstructive pulmonary disease (COPD), a disease with significant social, health, and economic burden. Therefore, we aimed to investigate the role of this factor in COPD exacerbation.

**Methods:**

We investigated 20‐ to 70‐year‐old patients who were admitted due to COPD exacerbation (acute phase) or presented to the pulmonary clinic for follow‐up (non‐acute phase). Correlation of uric acid and uric acid‐to‐creatinine ratio (UCR) with multiple factors and their predictive performance for more exacerbations and acute phase of COPD was investigated (receiver operating characteristic [ROC] analysis).

**Results:**

Overall, 63 patients were enrolled in this study, of whom 79.4% were men. Acute‐phase group encompassed 79.4% of the population with a greater rate of heavy smoking and average exacerbation in a year (*p*‐value = 0.009 and <0.001). The mean of uric acid and UCR was 5.6 (SD, 2.35) and 4.4 (SD, 1.9) in the total population, respectively, and were significantly higher in the acute phase and patients with frequent exacerbations (FE ≥ 3 exacerbations a year), *p*‐value <0.05. The area under the curve (AUC) of ROC analysis showed a high performance of uric acid and UCR for predicting acute phase (0.84 [95%CI, 0.73–0.96] and 0.86 [0.74–0.98]), FE (0.72 [0.60–0.85] and 0.75 [0.63–0.87]), and FE among acute‐phase patients (AUC, 0.63 [0.46–0.79] and 0.66 [0.50–0.81], respectively).

**Conclusion:**

Uric acid and UCR could be invaluable predictors of frequent exacerbation and the acute phase of COPD. Therefore, they might be applicable in evaluating the severity and progress of the disease.

## Graphical abstract

The graph shows the area under the curve (AUC) of ROC analysis, which used as a factor to illustrate the performance of uric acid and UCR for predicting frequent exacerbations of chronic obstructive pulmonary disease (COPD). According to this graph, uric acid and UCR could be invaluable measures in evaluating the severity and exacerbation of the disease.

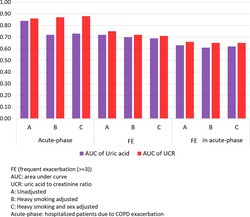

## 1 INTRODUCTION

Chronic obstructive pulmonary disease (COPD) is one of the most common and significant pulmonary diseases, with a global prevalence and mortality rate of 2744 and 42.4 per 100 000 persons.^1^ It is a chronic respiratory disease with zero to multiple exacerbations over a year that imposes a great social, health, and economic burden on patients' life.^2^, ^3^ Early detection of disease exacerbations could help to provide an earlier treatment and reduce the complications of the disease, such as cor pulmonale.^4^, ^5^ Multiple factors have been reported to be associated with frequent exacerbation and severity of the disease, such as forced expiratory volume in 1 s (FEV1), infection, and cigarette smoking.^6^, ^7^ One of the factors that have attracted attention in recent years is uric acid or uric acid‐to‐creatinine ratio level.^8^, ^9^

In case of frequent exacerbations and more severe COPD, prolonged hypoxemia develops that results in the destruction of adenosine, release of purine intermediates, and the end product of purine metabolism, such as uric acid. Therefore, serum uric acid has been suggested as a marker of oxidative metabolism disorder. Also, it is an independent prognostic factor of several cardiovascular disorders such as congestive pulmonary and heart failure, high blood pressure, and myocardial infarction.^10^ Given that, elevated creatinine level as the kidney functional factors might increase the level of uric acid, studies tried to control the confounding effect of creatinine by calculating the uric acid‐to‐creatinine ratio (UCR). In this order, multiple studies investigated the level of association between these factors and COPD severity.^8^, ^9^ Some of them indicated that uric acid and UCR might be valuable predictors of COPD severity and exacerbations.^3^, ^8^

Considering the great burden of the disease and the necessity for predicting exacerbations, we aimed to investigate the role of uric acid and UCR in COPD severity and exacerbation in hospitalized and no‐hospitalized patients. The results of this study can be used to determine the prognosis of COPD using a laboratory model.

## 2 MATERIALS AND METHODS

### 2.1 Study population

This cross‐sectional study enrolled COPD patients who were referred to Labafinejad Hospital of Tehran province in 2018 (Tehran is the capital city of Iran). Patients were selected from whom were referred to and admitted in the emergency with COPD exacerbation (50 patients) and pulmonary clinic (13 patients with stable COPD). Inclusion criteria were being 20–70 years old and diagnosed with COPD. Furthermore, patients with tuberculosis, lung cancer, interstitial lung disease, end‐stage renal disease, occupational disease, and immunosuppression were not included. The sampling days were randomly selected throughout the year. All patients were informed about the study and signed the informed consent.

### 2.2 Data gathering

All patients were interviewed, and the study data were gathered including demographics (age and sex), body mass index (BMI), smoking, history of chronic kidney disease (CKD), diabetes (DM), oxygen therapy at home (O_2_ therapy), dyspnea on exertion (DOE), and number of exacerbations in a year. Through physical examination, respiratory rate (RR), systolic blood pressure (SBP), diastolic blood pressure (DBP), and oxygen saturation (O_2_ sat) were measured. Afterward, blood sampling was done to obtain venous blood gases information, uric acid and creatinine level. UCR was calculated by dividing uric acid by creatinine level. The frequent exacerbations (FE) of the disease were defined as hospitalized people with greater than three exacerbations in a year. Patients hospitalized due to exacerbation were named the acute‐phase group, while patients with stable COPD who presented to the clinic were named the non‐acute‐phase group. Heavy smoking was defined as patients who smoked more than 20 pack‐year (p‐y) cigarettes. All data were collected in paper form. BMI was categorized into underweight (<18.5), healthy weight (18.5–24.9), and overweight (24.9<).^11^

### 2.3 Statistical analysis

After entering the data into an excel sheet, missing variables were gathered by calling the patients to receive missed information or reviewing their hospital documents. After cleaning the data, descriptive analysis was done using R version 3.5.2. Frequency tables describe the demographic factors of the patients, history of comorbidities, exacerbation frequency, FE, and laboratory results. Continuous variables including mean and standard deviation (SD) are reported. Categorical variables were presented in terms of number and percentage.

Afterward, independent samples T‐test and chi‐square were used to compare the frequency of different factors between sex groups and patients in acute‐phase versus non‐acute‐phase groups. Logistic regression was used for categorical variables with more than two categories. Then, the correlation between various variables with uric acid and UCR was estimated with linear regression, and the correlation coefficient was calculated with a 95% confidence interval (95%CI). The level of significance was considered as *p* < 0.05 throughout the study. Receiver operating characteristic (ROC) curves were used to evaluate the prognostic values of uric acid levels and UCR for predicting the FE and acute phase of the disease.

## 3 RESULTS

### 3.1 Descriptive

Overall, 63 patients were enrolled in this study, of whom 50 patients were men (79.4%). The mean age was 63.9 years (SD, 8.37). Fifty patients (79.4%) were in the acute‐phase group. Among all patients, 92.1% were smokers, and 61.9% reported more than 20 pack‐year smoking (Heavy smoking) were all males and no females were in this category. Moreover, it was significantly different between the acute‐phase and non‐acute‐phase groups (70.0% versus 30.8%; *p*‐value, 0.009). DOE was observed in 46% of the patients, significantly more in acute‐phase patients (*p*‐value < 0.001). Among all patients, 46% were on O_2_ therapy at home, and the mean of O_2_ saturation was 89.9% (SD, 5.15). On average, most of the patients had one or two exacerbations in a year (88.9%), while 7.9% reported ≥5 exacerbations; all were males and in the acute‐phase group. Also, 98% of acute‐phase and 53.8% of non‐acute‐phase groups had one or more exacerbations in the last year, respectively (*p*‐value, 0.001). FE (frequent exacerbation ≥ 3) was observed in 50% of the acute‐phase group and none of the non‐acute‐phase one. CKD and diabetes prevalence in our study population were 25.6% and 19.0%, respectively. Laboratory data showed that mean pCO_2_ level was 52.3 (SD, 11.0), significantly greater in males and acute‐phase patients (*p*‐value, 0.012 and <0.001, respectively) (Table 1).

**TABLE 1:** Descriptive analysis of the variables.

	Categories	Overall	Sex		*p*‐value	Disease phase						
			Female(*n* = 13)	Male(*n* = 50)		Non‐acute phase			Acute phase			*p*‐value (between total)
						Total(*n* = 13)	Female(*n* = 3)	Male(*n* = 10)	Total(*n* = 50)	Female(*n* = 10)	Male(*n* = 40)	
Age, mean (SD)	‐	63.90 (8.37)	65.54 (8.28)	63.48 (8.42)	0.43	58.77 (7.79)	64.67 (0.58)	57.00 (8.11)	65.24 (8.06)	65.80 (9.54)	65.10 (7.78)	**0.012**
Smoking (%)	0	5 (7.9)	5 (38.5)	0 (0.0)	**<0.001**	0 (0.0)	0 (0.0)	0 (0.0)	5 (10.0)	5 (50.0)	0 (0.0)	**0.003**
	<10 p‐y	6 (9.5)	5 (38.5)	1 (2.0)		4 (30.8)	3 (100.0)	1 (10.0)	2 (4.0)	2 (20.0)	0 (0.0)	
	10–20 p‐y	13 (20.6)	3 (23.1)	10 (20.0)		5 (38.5)	0 (0.0)	5 (50.0)	8 (16.0)	3 (30.0)	5 (12.5)	
	>20 p‐y	39 (61.9)	0 (0.0)	39 (78.0)		4 (30.8)	0 (0.0)	4 (40.0)	35 (70.0)	0 (0.0)	35 (87.5)	
Heavy smoking (%)	No	24 (38.1)	13 (100.0)	11 (22.0)	**<0.001**	9 (69.2)	3 (100.0)	6 (60.0)	15 (30.0)	10 (100.0)	5 (12.5)	**0.009**
	Yes	39 (61.9)	0 (0.0)	39 (78.0)		4 (30.8)	0 (0.0)	4 (40.0)	35 (70.0)	0 (0.0)	35 (87.5)	
Average exacerbations in a year (%)	0	7 (11.1)	2 (15.4)	5 (10.0)	0.61	6 (46.2)	2 (66.7)	4 (40.0)	1 (2.0)	0 (0.0)	1 (2.5)	**<0.001**
	1 and 2	31 (49.2)	6 (46.2)	25 (50.0)		7 (53.8)	1 (33.3)	6 (60.0)	24 (48.0)	5 (50.0)	19 (47.5)	
	3 and 4	20 (31.7)	5 (38.5)	15 (30.0)		0 (0.0)	0 (0.0)	0 (0.0)	20 (40.0)	5 (50.0)	15 (37.5)	
	≥5	5 (7.9)	0 (0.0)	5 (10.0)		0 (0.0)	0 (0.0)	0 (0.0)	5 (10.0)	0 (0.0)	5 (12.5)	
DM (%)	Yes	12 (19.0)	3 (23.1)	9 (18.0)	0.99	1 (7.7)	0 (0.0)	1 (10.0)	11 (22.0)	3 (30.0)	8 (20.0)	0.44
	No	51 (81.0)	10 (76.9)	41 (82.0)		12 (92.3)	3 (100.0)	9 (90.0)	39 (78.0)	7 (70.0)	32 (80.0)	
DOE (%)	Yes	46 (73.0)	8 (61.5)	38 (76.0)	0.49	3 (23.1)	0 (0.0)	3 (30.0)	43 (86.0)	8 (80.0)	35 (87.5)	**<0.001**
	No	17 (27.0)	5 (38.5)	12 (24.0)		10 (76.9)	3 (100.0)	7 (70.0)	7 (14.0)	2 (20.0)	5 (12.5)	
CKD (%)	Yes	16 (25.4)	4 (30.8)	12 (24.0)	0.89	3 (23.1)	1 (33.3)	2 (20.0)	13 (26.0)	3 (30.0)	10 (25.0)	1
	No	47 (74.6)	9 (69.2)	38 (76.0)		10 (76.9)	2 (66.7)	8 (80.0)	37 (74.0)	7 (70.0)	30 (75.0)	
O_2_ home (%)	Yes	29 (46.0)	7 (53.8)	22 (44.0)	0.78	3 (23.1)	1 (33.3)	2 (20.0)	26 (52.0)	6 (60.0)	20 (50.0)	0.12
	No	34 (54.0)	6 (46.2)	28 (56.0)		10 (76.9)	2 (66.7)	8 (80.0)	24 (48.0)	4 (40.0)	20 (50.0)	
BMI	Underweight	2 (3.17)	0 (0.00)	2 (4.00)	0.73	0 (0.00)	0 (0.00)	0 (0.00)	2 (4.00)	0 (0.00)	2 (5.00)	0.12
	Healthy Weight	16 (25.40)	3 (23.08)	13 (26.00)		1 (7.69)	0 (0.00)	1 (10.00)	15 (30.00)	3 (30.00)	12 (30.00)	
	Overweight	45 (71.43)	10 (76.92)	35 (70.00)		12 (92.31)	3 (100.00)	9 (90.00)	33 (66.00)	7 (70.00)	26 (65.00)	
SBP, mean (SD)	‐	12.84 (1.53)	13.15 (1.57)	12.76 (1.52)	0.41	12.77 (1.30)	12.00 (1.00)	13.00 (1.33)	12.86 (1.59)	13.50 (1.58)	12.70 (1.57)	0.85
DBP, mean (SD)	‐	7.84 (0.87)	7.77 (0.83)	7.86 (0.89)	0.75	8.00 (0.82)	7.33 (0.58)	8.20 (0.79)	7.80 (0.89)	7.90 (0.88)	7.77 (0.90)	0.46
O_2_ sat, mean (SD)	‐	89.87 (5.15)	87.92 (6.03)	90.38 (4.84)	0.13	90.46 (2.88)	90.00 (2.00)	90.60 (3.17)	89.72 (5.61)	87.30 (6.77)	90.33 (5.21)	0.65
RR, mean (SD)	‐	20.08 (8.90)	18.54 (3.15)	20.48 (9.84)	0.49	16.69 (1.55)	16.33 (1.53)	16.80 (1.62)	20.96 (9.78)	19.20 (3.26)	21.40 (10.81)	0.12
pH, mean (SD)	‐	7.37 (0.05)	7.37 (0.06)	7.37 (0.06)	0.95	7.38 (0.03)	7.38 (0.03)	7.38 (0.04)	7.37 (0.06)	7.37 (0.06)	7.37 (0.06)	0.53
HCO_3_, mean (SD)	‐	28.86 (6.58)	25.62 (4.17)	29.70 (6.86)	**0.045**	24.23 (2.28)	23.67 (3.51)	24.40 (2.01)	30.06 (6.81)	26.20 (4.34)	31.02 (7.01)	**0.004**
pCO_2_, mean (SD)	‐	52.25 (11.03)	45.54 (8.86)	54.00 (10.94)	**0.012**	42.85 (2.76)	41.67 (2.08)	43.20 (2.94)	54.70 (11.06)	46.70 (9.86)	56.70 (10.53)	**<0.001**
UCR, mean (SD)	‐	4.36 (1.87)	3.87 (1.73)	4.48 (1.90)	0.30	2.63 (1.24)	2.16 (0.37)	2.77 (1.38)	4.81 (1.75)	4.38 (1.64)	4.91 (1.78)	**<0.001**
Uric acid, mean (SD)	‐	5.60 (2.35)	5.16 (2.43)	5.72 (2.34)	0.45	3.55 (1.42)	2.43 (0.51)	3.88 (1.44)	6.14 (2.25)	5.98 (2.14)	6.18 (2.30)	**<0.001**

*Note*: Bold numbers show statistically significant *p*‐value.

Abbreviations: BMI, body mass index; CKD, chronic kidney disease; DM, diabetes mellitus; DOE, dyspnea in exertion; DPB, diastolic blood pressure; RR, respiratory rate; SBP, systolic blood pressure; UCR, uric acid‐to‐creatinine ratio.

**FIGURE 1:**
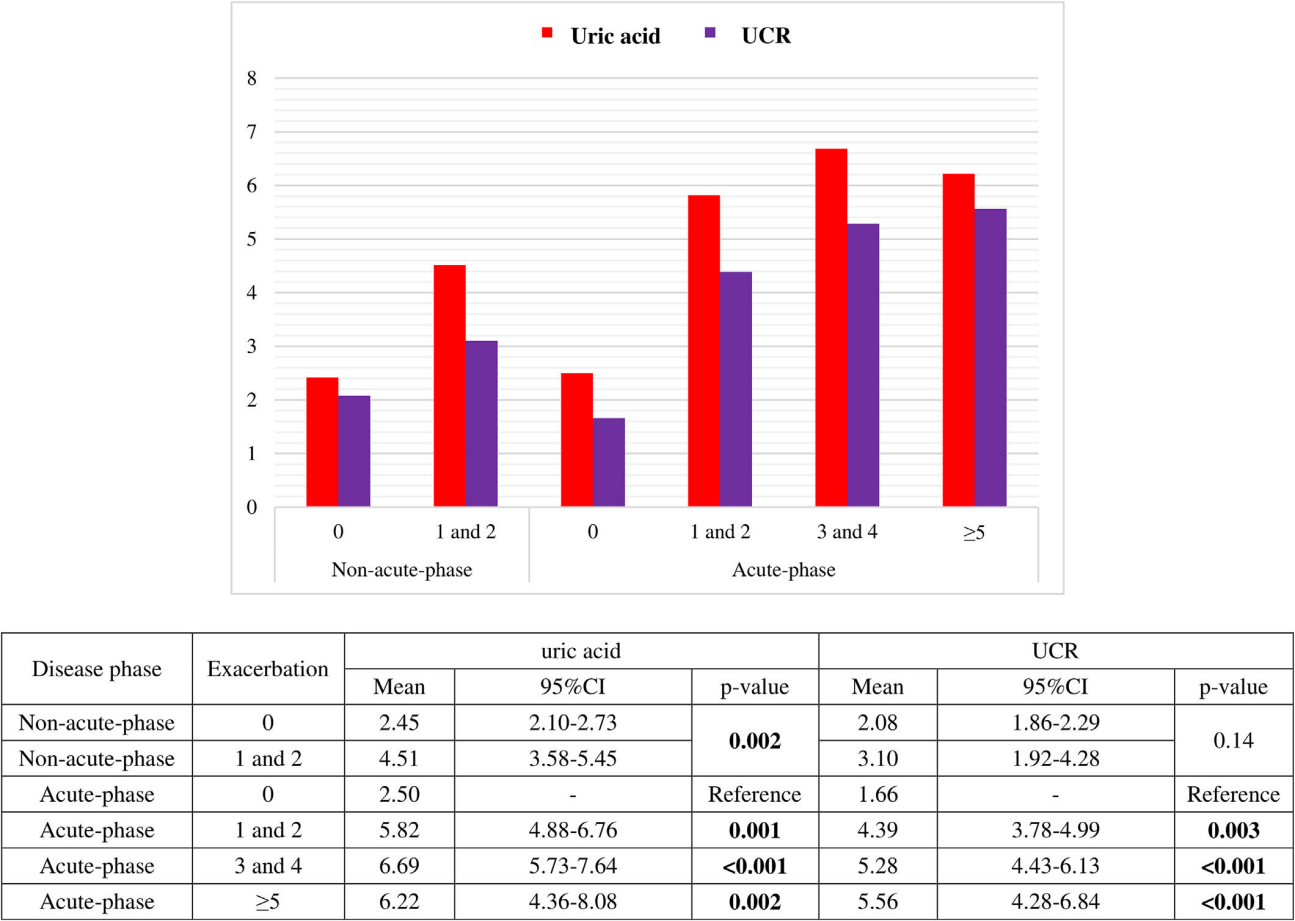
Mean of uric and UCR level based on different exacerbations in acute‐phase and non‐acute‐phase patients.

The mean of uric acid was 5.6 in the study population (SD, 2.35), which was significantly higher in the patients in acute phase, 3.55 (SD, 1.42) in the non‐acute phase versus 6.14 (SD, 2.25) in acute phase, *p*‐value < 0.001. Moreover, the mean of UCR was 4.4 (SD, 1.9) in the total population and 2.63 (SD, 1.24) and 4.81 (SD, 1.75) in acute‐phase and non‐acute‐phase groups, respectively (*p*‐value < 0.001). The mean of UCR was significantly greater in people with 1 or 2, 3 or 4, and 5≤ exacerbations in a year compared with the no exacerbations group: *p*‐values, 0.003, <0.001, and <0.001, respectively. People who declared to have DOE showed greater records of uric acid and UCR (*p*‐values < 0.001). The mean of SBP was negatively correlated with the mean of UCR: *p*‐value, 0.04, correlation coefficient, −0.32 (95%CI, −0.62, −0.02). However, O_2_ therapy at home was only correlated with greater uric acid (*p*‐value, 0.003). Moreover, a significantly greater mean of uric acid and UCR was observed in people with severe disease (*p*‐values, 0.006 and <0.001, respectively). Being in a heavy smoker group and older ages were only significantly correlated with uric acid level (*p*‐value, 0.04 and 0.01, respectively) although heavy smoker patients had a greater mean of UCR, it was not statistically significant; 3.84 (SD, 1.83) is the mean of UCR in non‐heavy smokers, versus 4.67 (SD, 1.85) in heavy smokers, *p*‐value = 0.09 (Table 2). Looking at acute‐phase and non‐acute‐phase patients, level of UCR was not significantly greater in patients with one or two exacerbations in a year compared with no exacerbations (all *p*‐values < 0.05) (Figure 1).

**TABLE 2:** Linear regression analysis of association of uric acid and UCR level with different variables.

Variables	Categories	Uric acid			UCR		
		Mean (SD)	Linear regression		Mean (SD)	Linear regression	
			Association coefficient	*p*‐value		Association coefficient	*p*‐value
Age	‐	‐	**0.09 (0.02, 0.16)**	**0.01**	‐	0.05 (−0.00, 0.1)	0.06
Sex	Female	5.16 (2.43)	Reference	0.45	3.87 (1.73)	Reference	0.29
	Male	5.72 (2.33)	0.56 (−0.91, 2.03)		4.48 (1.90)	0.61 (−0.55, 1.78)	
Smoking	0	6.82 (2.71)	Reference	‐	4.39 (1.85)	Reference	‐
	<10 p‐y	3.50 (1.67)	**−3.32 (−6.00, −0.64)**	**0.016**	3.32 (1.92)	−1.07 (−3.32, 1.18)	0.35
	10–20 p‐y	4.70 (1.83)	−2.12 (−4.45, 0.21)	0.074	3.87 (1.86)	−0.52 (−2.47, 1.44)	0.6
	>20 p‐y	6.07 (2.32)	−0.75 (−2.85, 1.36)	0.481	4.67 (1.85)	0.28 (−1.48, 2.05)	0.75
Average exacerbations in a year	0	2.43 (0.35)	Reference	‐	2.02 (0.28)	Reference	**‐**
	1 and 2	5.53 (2.17)	**3.10 (1.39, 4.81)**	**0.001**	4.10 (1.58)	**2.08 (0.74, 3.42)**	**0.003**
	3 and 4	6.69 (2.14)	**4.26 (2.46, 6.05)**	**<0.001**	5.28 (1.90)	**3.27 (1.86, 4.68)**	**<0.001**
	≥5	6.22 (2.08)	**3.79 (1.40, 6.18)**	**0.002**	5.56 (1.43)	**3.55 (1.67, 5.43)**	**<0.001**
DM	No	5.35 (2.41)	Reference	0.08	4.48 (1.88)	Reference	0.3
	Yes	6.66 (1.78)	1.30 (−0.18, 2.78)		3.85 (1.80)	−0.63 (−1.83, 0.57)	
DOE	No	**3.73 (1.67)**	Reference	**<0.001**	3.06 (1.38)	Reference	**<0.001**
	Yes	**6.30 (2.19)**	**2.56 (1.39, 3.73)**		4.83 (1.80)	**1.77 (0.80, 2.74)**	
CKD	No	**5.20 (2.32)**	**Reference**	**0.02**	‐	‐	**‐**
	Yes	**6.79 (2.07)**	**1.59 (0.28, 2.89)**		‐	**‐**	
O_2_ home	No	**4.81 (2.01)**	**Reference**	**0.003**	4.18 (1.70)	Reference	0.43
	Yes	**6.54 (2.40)**	**1.74 (0.62, 2.85)**		4.56 (2.07)	0.37 (−0.57, 1.32)	
BMI	Underweight	4.4 (0.84)	Reference	‐	5.22 (2.72)	Reference	‐
	Healthy Weight	5.62 (2.17)	1.22 (−2.35, 4.79)	0.50	4.63 (1.67)	−0.59 (−3.41, 2.24)	0.68
	Overweight	5.65 (2.47)	1.25 (−2.18, 4.69)	0.47	4.22 (1.93)	−1 (−3.72, 1.73)	0.47
SBP, mean (SD)	‐	‐	−0.04 (−0.44, 0.35)	0.83	‐	**−0.32 (−0.62, −0.02)**	**0.04**
DBP, mean (SD)	‐	‐	−0.39 (−1.07, 0.30)	0.26	‐	−0.49 (−1.03, 0.05)	0.07
O_2_ sat, mean (SD)	‐	‐	0.03 (−0.09, 0.14)	0.67	‐	0.04 (−0.05, 0.14)	0.35
RR, mean (SD)	‐	‐	0.06 (−0.003, 0.13)	0.06	‐	0.02 (−0.03, 0.07)	0.46
pH, mean (SD)	‐	‐	−4.26 (−15.20, 6.67)	0.44	‐	−0.27 (9.02, 8.48)	0.95
HCO_3_, mean (SD)	‐	‐	0.01 (−0.08,0.10)	0.84	‐	0.04 (−0.03,0.11)	0.26
pCO_2_, mean (SD)	‐	‐	0.03 (−0.03,0.08)	0.32	‐	0.04 (−0.003,0.08)	0.07
Disease phase	Non‐acute phase	**3.55 (1.42)**	**Reference**	**<0.001**	2.63 (1.23)	**Reference**	**<0.001**
	Acute phase	**6.14 (2.25)**	**2.59 (1.28, 3.91)**		4.81 (1.75)	**2.18 (1.15–3.21)**	
FE (frequent exacerbation [≥3])	No	**4.96 (2.30)**	**Reference**	**0.006**	3.71 (1.64)	**Reference**	**<0.001**
	Yes	**6.59 (2.09)**	**1.64 (0.49, 2.78)**		5.34 (1.79)	**1.62 (0.75, 2.50)**	
Heavy smoking	No	**4.84 (2.23)**	**Reference**	**0.04**	3.84 (1.83)	Reference	0.09
	Yes	**6.07 (2.32)**	**1.23 (0.05, 2.42)**		4.67 (1.85)	0.83 (−0.12, 1.78)	

*Note*: Bold numbers show statistically significant *p*‐value.

Abbreviations: BMI, body mass index; CKD, chronic kidney disease; DM, diabetes mellitus; DOE, dyspnea in exertion; DPB, diastolic blood pressure; RR, respiratory rate; SBP, systolic blood pressure; UCR, uric acid‐to‐creatinine ratio.

### 3.2 ROC analysis

**FIGURE 2:**
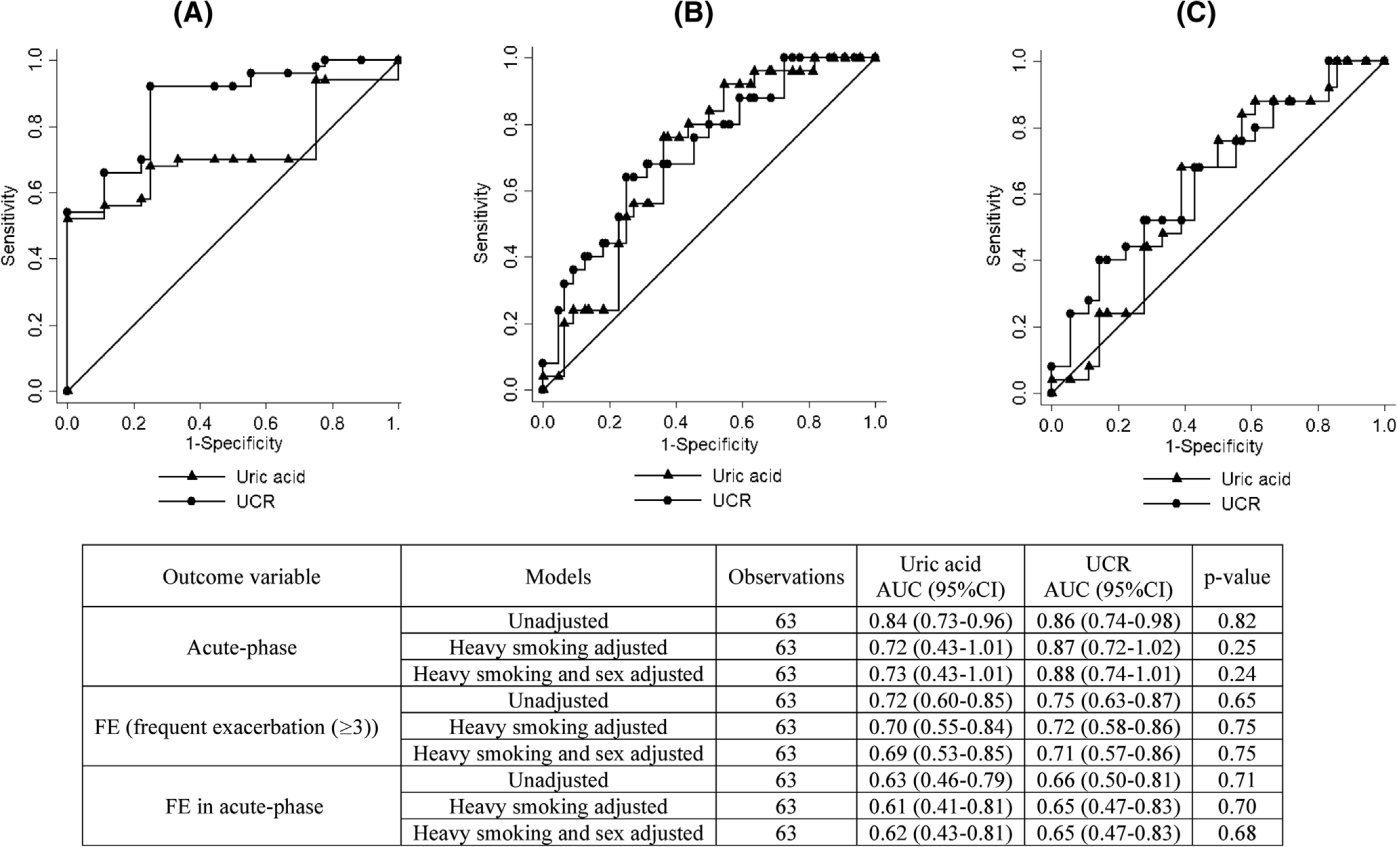
Smoking adjusted ROC curves for uric and URC by acute phase (A), FE (B), and FE in acute phase (C).

The area under the curve (AUC) obtained from the ROC analyses showed high performance of uric acid and UCR for predicting acute phase (0.84 [95%CI, 0.73–0.96], and 0.86 [0.74–0.98]) and FE (0.72 [0.60–0.85] and 0.75 [0.63–0.87], respectively). After adjusting the ROC analysis with heavy smoking, the AUC of uric acid to predict the acute phase changed to 0.72 [0.43–1.01]. The performance was lower for FE among acute‐phase patients (AUC, 0.63 [0.46–0.79] and 0.66 [0.50–0.81] for uric acid and UCR, respectively). The heavy smoking adjusted measures are presented in Figure 2. Our results indicated no significant difference between the performance of uric acid and UCR for predicting FE, acute phase, and FE in acute‐phase patients in the adjusted model: *p*‐value, 0.75, 0.25, and 0.70. The sensitivity and specificity of these indicators for predicting the parameters are summarized in Table 3.

**TABLE 3:** Sensitivity and specificity of uric acid and UCR for predicting acute phase, FE, and FE in acute‐phase patients.

Uric acid			UCR		
Acute phase			Acute phase		
Point	Sensitivity	Specificity	Point	Sensitivity	Specificity
2.0	100.0%	0.0%	1.7	100.0%	7.7%
2.6	98.0%	30.8%	2.0	98.0%	23.1%
3.0	96.0%	46.2%	2.1	96.0%	38.5%
3.5	92.0%	61.5%	2.6	92.0%	69.2%
4.0	84.0%	61.5%	2.7	92.0%	76.9%
4.5	76.0%	76.9%	2.8	90.0%	76.9%
5.0	66.0%	76.9%	4.0	70.0%	84.6%
5.5	58.0%	84.6%	4.2	66.0%	92.3%
6.0	54.0%	92.3%	5.0	46.0%	92.3%
7.1	36.0%	100.0%	5.5	34.0%	92.3%
8.0	18.0%	100.0%	6.0	26.0%	100.0%
8.9	10.0%	100.0%	7.1	10.0%	100.0%
14.0	2.0%	100.0%	10.7	2.0%	100.0%

Uric acid			UCR		
FE			FE		
Point	Sensitivity	Specificity	Point	Sensitivity	Specificity
2.0	100.0%	0.0%	1.7	100.0%	2.6%
2.6	100.0%	13.2%	2.0	100.0%	10.5%
3.0	100.0%	21.1%	2.1	100.0%	21.1%
3.5	100.0%	31.6%	2.6	100.0%	34.2%
4.0	96.0%	39.5%	2.7	100.0%	36.8%
4.5	88.0%	50.0%	2.8	100.0%	39.5%
5.0	80.0%	57.9%	4.0	80.0%	55.3%
5.5	72.0%	65.8%	4.2	76.0%	60.5%
6.0	64.0%	68.4%	5.0	52.0%	71.1%
7.1	44.0%	81.6%	5.5	44.0%	84.2%
8.0	12.0%	84.2%	6.0	36.0%	89.5%
8.9	8.0%	92.1%	7.1	16.0%	97.4%
14.0	4.0%	100.0%	10.7	4.0%	100.0%

Uric acid			UCR		
FE in acute‐phase group			FE in acute‐phase group		
Point	Sensitivity	Specificity	Point	Sensitivity	Specificity
2.5	100.0%	0.0%	1.7	100.0%	0.0%
3.0	100.0%	8.0%	2.0	100.0%	4.0%
3.5	100.0%	16.0%	2.1	100.0%	12.0%
4.0	96.0%	28.0%	2.7	100.0%	16.0%
4.5	88.0%	36.0%	2.8	100.0%	20.0%
5.0	80.0%	48.0%	3.3	88.0%	28.0%
5.6	72.0%	56.0%	4.0	80.0%	40.0%
6.1	60.0%	64.0%	4.2	72.0%	44.0%
7.1	44.0%	72.0%	5.0	52.0%	60.0%
7.6	28.0%	76.0%	5.5	44.0%	76.0%
8.1	8.0%	76.0%	6.0	36.0%	84.0%
10.0	4.0%	92.0%	7.1	16.0%	96.0%
14.0	4.0%	100.0%	10.7	4.0%	100.0%

Abbreviations: FE, frequent exacerbation (≥3); UCR, uric acid‐to‐creatinine ratio.

## 4 DISCUSSION

The results illustrated that uric acid and UCR levels are invaluable predictors of frequent COPD exacerbations and acute phase of the disease, without significant difference. The best result was observed in predicting the acute phase by UCR (AUC 0.86), although both UCR and uric acid also showed an AUC of more than 0.7 regarding predicting the FE and acute phase. Some other factors also demonstrated significant correlation with uric acid level, such as age, heavy smoking, and DOE (also with UCR), where heavy smoking influenced the correlation between uric acid and acute phase.

Uric acid and UCR were shown to be associated with worse COPD outcomes in previous studies.^8^, ^9^ Prolonged hypoxemia results in the destruction of adenosine, the release of purine intermediates, and the end product of purine metabolism, such as uric acid. Therefore, serum uric acid has been suggested as a marker of oxidative metabolism in the hypoxemic state, which develops in patients with more severe COPD and frequent exacerbations. On the other hand, uric acid might be increased in other disorders, such as renal failure, which is one of the comorbidities in COPD patients. Given that creatinine level is one of the renal failure indicators, the current study controlled the effect of renal failure on uric acid by considering the UCR as well as the uric acid level.^8^ Our results also revealed that uric acid and UCR levels are greater in patients with more COPD exacerbations.

Multiple studies have investigated the association of uric acid with frequent exacerbation of COPD patients, and some illustrated a positive correlation,^8^, ^9^, ^12^ but some showed a negative one.^13^ The diversity in the results could be due to the difference in study design and sample size. For instance, Bartziokas's study on acute exacerbated COPD patients indicated that uric acid is higher in patients with frequent exacerbation and correlation with prolonged hospital stays, need for non‐invasive ventilator therapy, and in‐hospital mortality of COPD hospitalized patients.^12^ Furthermore, the Kahnert's study that was on 1966 patients from the German COPD cohort COSYCONET demonstrated a positive association between uric acid and the burden of exacerbation. However, their network of relationships between parameters showed that reduced FEV1 is the intermediate variable of this correlation.^14^ Our study also revealed similar results regarding frequent exacerbation in total and hospitalized COPD patients, while we did not include spirometry parameters.

On the other hand, some other studies, such as Kocak et al.'s study that assessed uric acid in stable COPD patients, showed no significant correlation between frequent exacerbation and higher level of Global Initiative for Chronic Obstructive Lung Disease (GOLD).^8^ However, they implied that it might be a useful predictor of the abovementioned outcomes at higher cut‐offs. In their study, UCR was also investigated and compared with uric acid. They showed that AUC for uric acid and UCR was about 0.4 and 0.6 in predicting frequent exacerbation in stable COPD patients, respectively, which was lower than our findings in the total population but similar to UCR predicting exacerbation in acute‐phase patients (0.66). Even though the definition of frequent exacerbation differed between the two studies, we defined it as three or more exacerbations in a year, while it was two or more exacerbations in the other study. The other reason for the difference could be that our study population consisted of acute‐phase patients who have greater records of the parameters, while their study included stable COPD.^8^ It is noteworthy that although we investigated prediction level for three or more exacerbations in a year, our findings revealed that the presence of even one or two COPD exacerbations is associated with greater level of uric acid and UCR compared with people without exacerbation, except for UCR records of non‐acute‐phase (stable) patients.

One of the issues that we investigated in the current study is prediction values for different states of the disease by which we can hypothesize that uric acid and UCR are acceptable predictors of acute phase and hospitalization of COPD, with AUC of more than 0.7 and 0.8, respectively. Although we showed that uric acid and UCR might be acceptable predictors of frequent exacerbation, their AUC regarding acute phase or hospitalization is even more in particular for UCR. In this order, Bartziokas's study on acute exacerbated COPD patients showed that future hospitalization in 1‐year follow‐up was associated with greater uric acid levels.^12^ Also, another cohort study stated that a higher uric acid level is associated with hospitalization.^15^ Moreover, a case–control study on 283 stable COPD patients showed that uric and UCR are correlated with more last‐year hospitalization.^16^

Our results indicated no statistically significant difference between the performance of uric acid and UCR for predicting FE, acute phase, and FE in acute‐phase patients in the adjusted model. Even though the AUC of UCR was greater than uric acid in all prediction models, which was consistent with Kocak et al.'s study. Moreover, in a study of 59 stable COPD patients, UCR was correlated with FEV1, FVC, and dyspnea, while uric acid was only correlated with FVC.^17^ In another study on 109 stable COPD patients also, exacerbations in the last year was associated with a greater level of UCR but not uric acid.^9^ It was also illustrated in AbdelHalim's study on 283 patients that UCR was more powerful in predicting frequent exacerbation than uric acid.^16^ The significance of associations was not compared in any of the abovementioned studies, while we investigated it, which resulted in a not statistically significant difference between UCR and uric acid. Furthermore, all of the above studies were performed on stable patients, while we included acute‐phase ones.

Age was only correlated with uric acid level. However, its correlation with UCR was marginally insignificant (*p*‐value = 0.06), and an increase in the number of patients might differ the results. Overall, the severity of COPD as a slowly progressive airflow obstruction disease rises in older ages. In this order, age has been announced as one of the hospitalizations and reduced survival factors in these patients.^18^, ^19^ Similarly, heavy smoker patients had a greater mean of uric acid and UCR, which was only statistically significant regarding uric acid. Smoking, as the main exogenous factor of COPD, was the third main predictor of exacerbation frequency in AbdelHalim's study after UCR and uric acid.^16^ Their prediction model explained 88% of exacerbation frequency with a *p*‐value < 0.001, even though it was stated that smoking is correlated with a lower amounts of uric acid and UCR in another study.^20^ Furthermore, some studies investigated the role of amount, duration, and current status of smoking on uric acid level; one of them showed that current smoker COPD patients had significantly lower uric acid and UCR levels in comparison with non‐smokers and previous smoker patients.^9^ Regarding the correlation between smoking and uric acid level, several mechanisms have been investigated. In one view, current smoking is associated with increased ROS, which is a potent oxidant. Given that uric acid is one of the antioxidants acting against ROS, its concentration might decrease in long‐term exposure to smoking and ROS.^21^, ^22^ On the other hand, long‐term smoking has been shown to have some nephrotoxic effects that could result in higher levels of uric acid.^21^ Therefore, the correlation between smoking and uric acid is bidirectional, and the equilibrium between these mechanisms results in the final uric acid level. Furthermore, we showed that smoking is not influencing the predicting value of UCR, which could be explained by the mechanism that positive correlation between smoking and uric acid might be partly due to the impaired renal function, which does not affect UCR level (UCR is not influenced by renal function).

Although diet (intake of purines), BMI, and exercise were correlated with the number of purines and higher uric acid, we did not find a significant correlation between BMI and uric acid level.^8^ However, we did not investigate the role of high‐purine diet, which should be considered in further studies. Furthermore, serum uric acid, FEV1%, and 6‐MWD were reported to be dependent on BMI, which is associated with exacerbation.^14^ This difference is probably due to the sample size and variation in other patient factors.

Our findings revealed that O_2_ therapy at home (LTOT) was associated with greater levels of uric acid but not with UCR. A previous study on 91 outpatients with COPD who followed for 31 months and were on LTOT showed that change in UCR is predictive of long‐term mortality.^23^ We did not investigate changes in UCR and most of the study population were in acute phase, which could be the reasons for the difference.

One of the limitations of the study was not having access to spirometry analysis of the patients at the time of measuring uric acid, which needed to be addressed in further studies. However, spirometry features do not seem to influence the result of the study since they are an intermediate variable of the correlation between uric acid and COPD exacerbation.^14^ The other limitation was the low number of stable COPD patients who agreed to participate in the study. In the future studies, it is invaluable to investigate the reason for not participating in the study. Also, the most of the data about gradations of dyspnea as per Modified Medical Research Council (MMRC) was missing in the patients' records. Therefore, we were not able to categorize dyspnea.

## 5 CONCLUSION

We illustrated that uric acid and UCR could be invaluable predictors of frequent exacerbation and acute phase and could be used as indicators to evaluate the severity and progress of the disease in COPD patients. Although UCR showed greater measures in predicting all observed outcomes, it did not significantly differ from uric acid. Smoking is the main factor that affected the correlation between uric acid and the acute phase, which should be considered if uric acid is going to get involved in predicting or scoring system of COPD patients.

## AUTHOR CONTRIBUTIONS

The authors confirm contribution to the paper as follows: Saman Barmehziar, Abbas Fadaei, and Fariba Samadian designed the study. Sogol Koolaji, Saman Barmehziar, Fariba Samadian, and Ali Shakiba analyzed and interpreted the data. Sogol Koolaji, Saman Barmehziar, Abbas Fadaei, and Ali Shakiba drafted the manuscript. Sogol Koolaji, Abbas Fadaei, Saman Barmehziar, and Fariba Samadian revised the manuscript critically for important intellectual content. All authors read and approved the final manuscript.

## CONFLICT OF INTEREST STATEMENT

Authors declared no conflict of interests.

## ETHICS STATEMENT

This study was ethically approved by the Ethical Committee of Shahid Beheshti University of Medical Sciences (ID; IR.SBMU.MSP.REC.1398.280).

## Data Availability

The data that support the findings of this study are available from corresponding author upon reasonable request.

## References

[1] IHME . GBD Compare‐Viz Hub. 2022. https://collab2019.healthdata.org/gbd%2Dcompare/?utm%5Fsource%3DActive%2BCollaborators%26utm%5Fcampaign%3D7b019ca2ad%2DEMAIL%5FCAMPAIGN%5F2020%5F04%5F24%5F06%5F07%5FCOPY%5F01%26utm%5Fmedium%3Demail%26utm%5Fterm%3D0%5Fd65e2ca485%2D7b019ca2ad%2D422599137

[2] Seemungal TA , Donaldson GC , Paul EA , Bestall JC , Jeffries DJ , Wedzicha JA . Effect of exacerbation on quality of life in patients with chronic obstructive pulmonary disease. Am J Respir Crit Care Med. 1998;157(5):1418‐1422. doi:10.1164/ajrccm.157.5.9709032 9603117

[3] Garcia‐Aymerich J , Farrero E , Felez M , Izquierdo J , Marrades R , Anto J . Risk factors of readmission to hospital for a COPD exacerbation: a prospective study. Thorax. 2003;58(2):100‐105. doi:10.1136/thorax.58.2.100 12554887 PMC1746561

[4] Donaldson G , Wedzicha J . COPD exacerbations·1: epidemiology. Thorax. 2006;61(2):164‐168. doi:10.1136/thx.2005.041806 16443707 PMC2104564

[5] Kawut SM , Poor HD , Parikh MA , et al. Cor pulmonale parvus in chronic obstructive pulmonary disease and emphysema. J am Coll Cardiol. 2014;64(19):2000‐2009. doi:10.1016/j.jacc.2014.07.991 25440095 PMC4347835

[6] Wedzicha JA , Donaldson GC . Exacerbations of chronic obstructive pulmonary disease. Respir Care. 2003;48(12):1204‐1215. 14651761

[7] Celli BR , Cote CG , Marin JM , et al. The body‐mass index, airflow obstruction, dyspnea, and exercise capacity index in chronic obstructive pulmonary disease. N Engl J Med. 2004;350(10):1005‐1012. doi:10.1056/NEJMoa021322 14999112

[8] Kocak ND , Sasak G , Akturk UA , et al. Serum uric acid levels and uric acid/creatinine ratios in stable chronic obstructive pulmonary disease (COPD) patients: are these parameters efficient predictors of patients at risk for exacerbation and/or severity of disease? Med Sci Monit: Int Med J Exp Clin Res. 2016;22:4169‐4176. doi:10.12659/MSM.897759 PMC5098926 27811831

[9] Rumora L , Hlapčić I , Popović‐Grle S , Rako I , Rogić D , Čepelak I . Uric acid and uric acid to creatinine ratio in the assessment of chronic obstructive pulmonary disease: potential biomarkers in multicomponent models comprising IL‐1beta. PLoS ONE. 2020;15(6):e0234363. doi:10.1371/journal.pone.0234363 32502184 PMC7274385

[10] Feig DI , Kang D‐H , Johnson RJ . Uric acid and cardiovascular risk. N Engl J Med. 2008;359(17):1811‐1821. doi:10.1056/NEJMra0800885 18946066 PMC2684330

[11] Division of Nutrition PA, and Obesity, National Center for Chronic Disease Prevention and Health Promotion . About Adult BMI 2023. https://www.cdc.gov/healthyweight/assessing/bmi/adult_bmi/index.html

[12] Bartziokas K , Papaioannou AI , Loukides S , et al. Serum uric acid as a predictor of mortality and future exacerbations of COPD. Eur Respir J. 2014;43(1):43‐53. doi:10.1183/09031936.00209212 23645404

[13] Nicks ME , O'Brien MM , Bowler RP . Plasma antioxidants are associated with impaired lung function and COPD exacerbations in smokers. COPD: J Chron Obstruct Pulmon Dis. 2011;8(4):264‐269. doi:10.3109/15412555.2011.579202 PMC4486075 21627570

[14] Kahnert K , Alter P , Welte T , et al. Uric acid, lung function, physical capacity and exacerbation frequency in patients with COPD: a multi‐dimensional approach. Respir Res. 2018;19(1):110. doi:10.1186/s12931-018-0815-y 29866121 PMC5987642

[15] Vafaei A , Saremi Z , Mortazavi Moghaddam SG , Javid AZ . The relationship between serum uric acid and severity of chronic obstructive pulmonary disease (COPD). J Cardio‐Thorac Med. 2017;5(3):181‐186.

[16] AbdelHalim HA , AboElNaga HH . Serum uric acid levels and uric acid/creatinine ratios: affordable biomarkers for predicting chronic obstructive pulmonary disease severity and exacerbations. Egypt J Chest Dis Tuberc. 2018;67(3):231. doi:10.4103/ejcdt.ejcdt_39_18

[17] Garcia‐Pachon E , Padilla‐Navas I , Shum C . Serum uric acid to creatinine ratio in patients with chronic obstructive pulmonary disease. Lung. 2007;185(1):21‐24. doi:10.1007/s00408-006-0076-2 17294336

[18] Terzano C , Conti V , Di Stefano F , et al. Comorbidity, hospitalization, and mortality in COPD: results from a longitudinal study. Lung. 2010;188(4):321‐329. doi:10.1007/s00408-009-9222-y 20066539

[19] Siafakas N , Vermeire P , Na P , et al. Optimal assessment and management of chronic obstructive pulmonary disease (COPD). The European Respiratory Society Task Force. Eur Respir J. 1995;8(8):1398‐1420. doi:10.1183/09031936.95.08081398 7489808

[20] Hanna BE , Hamed JM , Touhala LM . Serum uric acid in smokers. Oman Med J. 2008;23(4):269‐274. 22334840 PMC3273920

[21] Kim S‐K , Choe J‐Y . Association between smoking and serum uric acid in Korean population: data from the seventh Korea national health and nutrition examination survey 2016. Medicine. 2019;98(7):e14507. doi:10.1097/MD.0000000000014507 30762781 PMC6407981

[22] Tsuchiya M , Asada A , Kasahara E , Sato EF , Shindo M , Inoue M . Smoking a single cigarette rapidly reduces combined concentrations of nitrate and nitrite and concentrations of antioxidants in plasma. Circulation. 2002;105(10):1155‐1157. doi:10.1161/hc1002.105935 11889006

[23] Sato N , Kurashima K , Ubukata M , et al. Prognostic significance of serum uric acid in patients with chronic obstructive pulmonary disease receiving home oxygen therapy. Nihon Kokyuki Gakkai Zasshi= the Journal of the Japanese Respiratory Society. 2003;41(2):74‐80. 12722324

